# Supplementary material for: An intersectional analysis of long COVID prevalence
Source: Int J Equity Health. 2023 Dec 13;22:261. doi: 10.1186/s12939-023-02072-5 (PMC10717295; doi:10.1186/s12939-023-02072-5)
Supplement: Supplementary file 1 — Supplementary Material 1 [file 12939_2023_2072_MOESM1_ESM.docx]

Appendix Table 1.

Sample Means and Cell Sizes

|  | Adults who had Covid | | Adults who had long Covid | |
| --- | --- | --- | --- | --- |
|  | Proportion | Number of respondents | Proportion | Number of respondents |
| Has long Covid | 0.28 | 79,401 | 1.00 | 59,166 |
| Has activity limitations | 0.10 | 27,678 | 0.36 | 21,427 |
| Has 4-yr degree | 0.56 | 160,745 | 0.46 | 27,229 |
| Female | 0.59 | 167,784 | 0.68 | 40,447 |
| Sexual/gender minority | 0.12 | 33,714 | 0.15 | 8,875 |
| Race/ethnicity |  |  |  |  |
| White | 0.75 | 215,499 | 0.74 | 43,567 |
| Hispanic | 0.10 | 27,826 | 0.11 | 6,700 |
| Black non-Hispanic | 0.06 | 18,239 | 0.07 | 4,075 |
| Asian non-Hispanic | 0.04 | 12,285 | 0.03 | 1,663 |
| Other race non-Hispanic | 0.04 | 11,900 | 0.05 | 3,161 |
| Age | 49.32 | 285,749 | 48.82 | 59,166 |
| Two-way interaction terms |  |  |  |  |
| Female*White | 0.44 | 124,329 | 0.49 | 29,191 |
| Female*Hispanic | 0.06 | 17,130 | 0.08 | 4,733 |
| Female*Black | 0.05 | 13,054 | 0.06 | 3,318 |
| Female*Asian | 0.02 | 5,905 | 0.02 | 981 |
| Female*Other race | 0.03 | 7,366 | 0.04 | 2,224 |
| Female*Has BA/BS | 0.32 | 90,172 | 0.30 | 17,955 |
| Female*SGM | 0.07 | 21,116 | 0.11 | 6,319 |
| Has BA/BS*White | 0.44 | 125,759 | 0.35 | 20,634 |
| Has BA/BS*Hispanic | 0.04 | 11,738 | 0.04 | 2,514 |
| Has BA/BS*Black | 0.03 | 8,156 | 0.03 | 1,646 |
| Has BA/BS*Asian | 0.03 | 9,609 | 0.02 | 1,180 |
| Has BA/BS*Other race | 0.02 | 5,483 | 0.02 | 1,255 |
| Has BA/BS*SGM | 0.07 | 18,837 | 0.07 | 4,242 |
| SGM*White | 0.08 | 24,137 | 0.11 | 6,298 |
| SGM*Hispanic | 0.02 | 4,330 | 0.02 | 1,239 |
| SGM*Black | 0.01 | 1,836 | 0.01 | 414 |
| SGM*Asian | 0.00 | 1,256 | 0.00 | 231 |
| SGM*Other race | 0.01 | 2,155 | 0.01 | 693 |
| Three-way interaction terms |  |  |  |  |
| Female*Has BA/BS*SGM | 0.04 | 11,288 | 0.05 | 2,930 |
| Female*Has BA/BS*White | 0.25 | 70,066 | 0.23 | 13,415 |
| Female*Has BA/BS*Hispanic | 0.02 | 6,794 | 0.03 | 1,712 |
| Female*Has BA/BS*Black | 0.02 | 5,578 | 0.02 | 1,299 |
| Female*Has BA/BS*Asian | 0.02 | 4,472 | 0.01 | 676 |
| Female*Has BA/BS*Other race | 0.01 | 3,262 | 0.01 | 853 |
| SGM*Has BA/BS*White | 0.05 | 14,229 | 0.05 | 3,144 |
| SGM*Has BA/BS*Hispanic | 0.01 | 1,927 | 0.01 | 508 |
| SGM*Has BA/BS*Black | 0.00 | 821 | 0.00 | 168 |
| SGM*Has BA/BS*Asian | 0.00 | 910 | 0.00 | 157 |
| SGM*Has BA/BS*Other race | 0.00 | 950 | 0.00 | 265 |
| Female*SGM*White | 0.05 | 15,074 | 0.08 | 4,465 |
| Female*SGM*Hispanic | 0.01 | 2,673 | 0.01 | 860 |
| Female*SGM*Black | 0.00 | 1,271 | 0.01 | 332 |
| Female*SGM*Asian | 0.00 | 647 | 0.00 | 150 |
| Female*SGM*Other race | 0.01 | 1,451 | 0.01 | 512 |
| Sample Size | 285,749 | 285,749 | 59,166 | 59,166 |
